# Functionalized Fiber End Superstructure Fiber Bragg Grating Refractive Index Sensor for Heavy Metal Ion Detection

**DOI:** 10.3390/s18061821

**Published:** 2018-06-05

**Authors:** Rex Xiao Tan, Stephanie Hui Kit Yap, Yung Chuen Tan, Swee Chuan Tjin, Morten Ibsen, Ken Tye Yong, Wenn Jing Lai

**Affiliations:** 1 School of EEE, Nanyang Technological University, 50 Nanyang Avenue, Singapore 639798, Singapore; step0031@e.ntu.edu.sg (S.H.K.Y.); esctjin@ntu.edu.sg (S.C.T.); ktyong@ntu.edu.sg (K.T.Y.); 2 Temasek Laboratories@NTU, 50 Nanyang Avenue, Research Techno Plaza, Singapore 637553, Singapore; yungchuen@ntu.edu.sg (Y.C.T.); wennjing@ntu.edu.sg (W.J.L.); 3 Optoelectronics Research Centre, University of Southampton, Southampton SO17 1BJ, UK; mi@orc.soton.ac.uk

**Keywords:** fiber sensors, fiber Bragg gratings, chemosensors

## Abstract

We present a novel superstructure fiber Bragg grating fiber end sensor capable of detecting variations in refractive index (RI) of liquids and potentially that of gases, and demonstrated an application in the detection of heavy metal ions in water. The sensor is capable of sensing RI variations in the range of 1.333 to 1.470 with good sensitivity of up to 230 dB/RIU achieved for the RI range of 1.370 to 1.390. The sensor is capable of simultaneously measuring variations in ambient temperature along with RI. A simple chemical coating was employed as a chelating agent for heavy metal ion detection at the fiber end to demonstrate an possible application of the sensor. The coated fiber sensor can conclusively detect the presence of heavy metal ions with concentrations upwards of 100 ppm. RI sensing capability of the sensor is neither affected by temperature nor strain and is both robust and easily reproducible.

## 1. Introduction

In recent years, optical fiber sensors have received a considerable amount of attention due to their advantages over their electronics counterparts. Fiber sensors are immune to electromagnetic interferences, compactible and able to withstand harsh environments without the need to employ excessive protection measures. Refractive Index (RI) sensing is one of the main interests amongst the substantial body of optical fiber works that have been reported to date. Many fiber technologies have been applied for RI sensing such as the micro fiber, which can be fabricated by etching [1] or tapering [2], and their fiber Bragg grating (FBG) inscribed versions [3]. Other technologies include long period fiber gratings [4] and dielectric material-coated sensors that induce surface plasmon resonances [5]. One common characteristic of these sensing schemes is the reduction in physical dimensions of the cladding or core to expose the core guided modes to the environment. Although these processes do greatly enhance the sensitivity of these fiber sensors, they inevitably decrease the mechanical strength of the host fiber, resulting in complications that can be effected by variations in ambient temperature and environmentally induced strains on the fiber.

Much interest has been shown in FBG sensors as exemplified by the multitude of research on them. Variations in wavelength of the back-reflected light observed when these FBGs experience elastic (plastic) deformation due to the presence of various environmental stressors have led to several studies into the potential applications of FBGs as thermocouples [6] and strain gauges [7,8]. Light propagating through the grating and its vicinity does not, however, interact with the ambient environment in any way except in cases of a tilted fiber Bragg grating sensors and this has limited the RI sensing capabilities of FBGs.

To overcome this limitation, tilted FBGs (TFBG) [9,10,11,12,13] were employed. Also reported was a ratio-meter style fiber end RI sensor [14,15] using FBG reflection as a reference. The sensing mechanism of a TFBG is based on the out-coupling of backward-propagating cladding modes as the RI of the environment changes. Interrogation of TFBGs can be carried out through monitoring of the shift in wavelengths or variations in power of the spectral features formed by the backward propagating cladding modes. However, fabrication of TFBGs may pose some challenges as it is not easy to accurately align and hence difficult to write repeatable long TFBGs. Extensive demodulation methods and data processing may also be required to measure the power of cladding modes over a range of wavelengths.

On the other hand, various methods such as reducing the diameter of the fiber cladding through chemical etching [16,17] or inscribing the grating in the fiber cladding [18] have also been explored. Lastly, sensing schemes utilizing a Fabry–Perot interferometer (FPI) configuration [19,20] have been proposed. Fiber end sensors such as the FBG-FPI work presented in [19,20] are generally free from the aforementioned limitations of weakened physical structure and temperature and strain complications, proving to be viable RI sensing probes. These sensors are fabricated through the inscription of a FBG into an optical fiber and cleaving the optical fiber at a short distance from the FBG. This results in the formation of an FPI, with the FBG and the cleaved end of the fiber behaving as the reflecting surfaces. Variations in RI of the ambient environment would result in a variation in the reflectance of light at the cleaved end. This would then be registered as a variation in visibility (*v*) of the interference fringes in the output spectrum with the FBG reflection serving as a reference point. This way of interrogation eliminates the need for the incorporation of an additional reference arm [21] in the sensing setup. Coupled with its low fabrication complexity and the retaining of its mechanical strength (as compared to sensing probes which have had a portion of the fiber structure removed), the FBG-FPI is a highly viable sensing probe for various industrial sensing applications.

Environmental agencies around the world are also particularly interested in RI sensors that can detect specific pollutants such as heavy metals in bodies of water. The presence of these heavy metal ions would lead to a variation in RI of the body of water. However, at low concentrations (parts per million (ppm) levels), these heavy metals (outside of laboratory conditions) are difficult to detect and do not result in any detectable variations in RI by regular refractometers. Optical fiber-based RI sensors focusing on this problem have been previously developed by our group [22], but these sensors were found to be cross-sensitive to temperature and strain as well.

In this paper, we present a superstructure fiber Bragg grating (SSFBG) fiber end refractive index sensor based on the previously mentioned FBG-FPI for RI sensing applications but with a SSFBG for larger sensing range due to the SSFBG’s reflection characteristics comb like multiple reflection peaks. The SSFBG was written on two different fibers with different effective indices and both sensors exhibited good RI sensing characteristics in the RI range of 1.333 to 1.470. Both sensors were then coated with Ethylenediaminetetraacetic acid (EDTA), which enabled them to detect the presence of heavy metal ions upwards of 100 ppm. As compared to other sensing schemes, this SSFBG-FPI sensor is comparable to tilted FBG schemes in terms of sensitivity and working range but with a simpler interrogation requirement, superior to modified fiber cladding sensors in sensor robustness and higher in sensitivity as compared to ratio-meter configurations, which is prone to influence from noise.

## 2. Theory

### 2.1. Fiber End Reflection

The Fresnel reflection of light at the cleaved end of a fiber is a function of the mismatch between the fiber effective index and that of the ambient environment and can be expressed as:

(1) $$R\approx \frac{{\left(n_{eff}-n_{ambient}\right)}^{2}}{{\left(n_{eff}+n_{ambient}\right)}^{2}},$$

where $n_{eff}$ is the fiber effective RI as seen by the fundamental guided mode in the optical fiber and $n_{ambient}$ is the ambient RI in which the cleaved fiber end is directly exposed to. In this work, the PS1250 fiber ($n_{eff}$ = 1.448 at around 1520 nm) from Fibercore (Las Vegas, NV, USA) and UHNA7 fiber ($n_{eff}$ = 1.478 at around 1520 nm) from Nufern (East Granby, CT, USA) were used. Under standard conditions, the refractive index of air ($n_{env}$ in this case) is approximately 1.002. From here, the percentage of fiber end reflection in air for the PS1250 and the UHNA7 can be estimated to be approximately 3.3% and 3.7%, respectively. As can be seen from Equation (1), a variation in $n_{ambient}$ either through gas pressurization and heating or immersion of the fiber end in liquids with different RIs would result in a variation in end reflection. The direct monitoring of this reflected optical power can be used for RI sensing [14] but is susceptible to errors due to power fluctuations of the light source or bending of the fiber in any part of the optical system.

### 2.2. Superstructure Fiber Bragg Grating

FBGs are periodic structures inscribed in the core of a fiber, typically by means of periodic refractive index modulation achieved through Ultraviolet (UV) irradiation [23]. Light propagating through the core of the fiber would, as a consequence of these periodic perturbations, can be back-reflected along the core at a specific wavelength (Bragg wavelength, $\lambda_{Bragg}$) with a certain linewidth. The Bragg wavelength would depend on the period (Λ) of the perturbations and effective index ($n_{eff}$) of the optical fiber:

(2) $$\lambda_{Bragg}=2n_{eff}\Lambda .$$

SSFBGs are special FBGs that have periodic index modulations distributed across a segment of fiber in the fiber core. This is effectively a secondary index profile modulations in a length of uniform fiber Bragg grating with a much longer pitch than the Bragg grating itself. The structure can be introduced during writing of the grating using various methods such as the amplitude mask over a phase mask method described in this paper. The reflectivity characteristics of such structures are predicted to present reflection peaks from each spatial Fourier component of the index perturbation.

Hence, despite being a single grating, the effect of a SSFBG is similar to several smaller FBGs placed close to each other. The resulting reflection spectrum consists of multiple peaks at different frequencies with different reflectivity and other higher order Fourier components [23,24]. FBGs or SSFBGs by themselves are not sensitive to perturbations in ambient RI unless they are inscribed in an etched or a tapered fiber. However, when the grating forms a Fabry–Perot (FP) cavity with optical fiber end reflection that is directly affected by the environment, an FPI is formed at Bragg wavelengths dictated by the grating parameters. The key advantage of a SSFBG-Fiber end reflection RI sensor over other FBG-Fiber end reflection RI sensors is its wide RI sensitivity range and resolvability due to the reflection characteristics of the SSFBG discussed in detail in Section 2.3. Similar to regular FBGs, SSFBGs can be thermally tuned. This allows the SSFBG to also be used as a temperature sensor. The response of a single FBG to fluctuations in ambient temperature [25] is well studied and is given by:

(3) $$\frac{\Delta \lambda_{Bragg}}{\lambda_{Bragg}}=\left(\alpha_{\Lambda }+\alpha_{n}\right)\Delta T,$$

where $\alpha_{\Lambda }$ the thermal expansion coefficient of the optical fiber and $\alpha_{n}$ is the thermo-optic coefficient. As such, when the FBG is subjected to temperature perturbations, the Bragg wavelength would experience a red or blue shift due to an increase or decrease in temperature, respectively.

### 2.3. SSFBG-Fiber End Fabry–Perot Cavity

**Figure 1:**
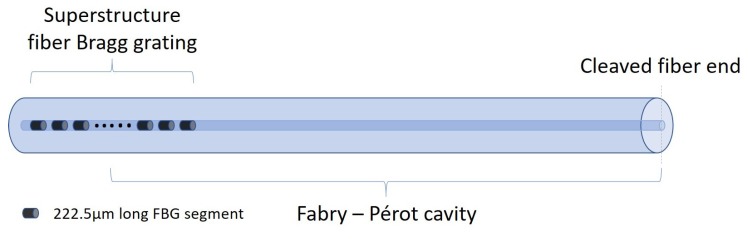
SSFBG-Fiber cleaved end Fabry–Perot cavity.

A low finesse cavity formed by a SSFBG and the cleaved fiber end (Figure 1) is the basis of the demonstrated sensors. The Fabry–Perot interference in the sensors are formed between this characteristic reflection spectrum of the SSFBG discussed in Section 2.2 and the broadband cleaved fiber end reflection. Variation in pitch of the gratings and their effect on the output spectra and sensor response will be the subject of future studies.

**Figure 2:**
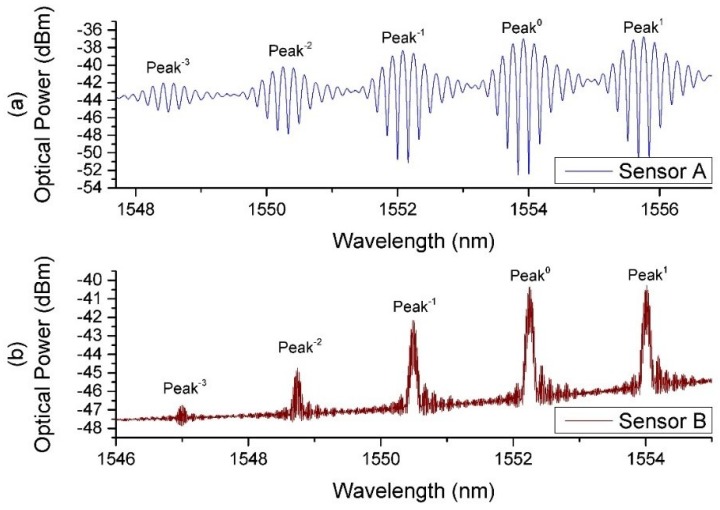
SSFBG-Fiber cleaved end Fabry–Perot interference pattern of (**a**) Sensor A and (**b**) Sensor B.

The spectrum of the SSFBG-Fiber end Fabry–Perot cavities fabricated using the PS1250 (Sensor A) and the UHNA7 (Sensor B) fibers are shown in Figure 2.

For ease of explanation, we have labeled the center SSFBG peak at 1554.1 nm and 1552.3 nm as peak 0 for this work for Sensors A and B, respectively. It was also established that only peaks −3 to peak 1 for both sensors were necessary for providing the required reflection power difference between peaks for the desired sensing applications. As characteristic of SSFBGs, peaks are spaced at regular spectral intervals with decreasing reflective power as the distance from central reflection peak increases.

It is obvious that Sensors A and B exhibit noticeably different spectral patterns. This is attributed to the fact that the sensors were hosted in different fiber types, doped with different dopants and have different core diameters. These factors resulted in a difference in photosensitivity and fundamental mode field diameter (MFD) between the two host fibers. Sensor A fiber PS1250 is Germanium and Boron co-doped, designed specifically with a photosensitivity while Sensor B fiber UHNA 7 is doped with high concentration of Germanium, designed as a high NA fiber with lower photosensitivity in comparison. Therefore, to match the FBG reflectivity closely to the respective end reflectivity, which is 3.3% for PS1250 and 3.7% for UHNA7, a longer grating had to be inscribed for Sensor B. Actual parameters of the sensors are discussed in Section 3.1.

As grating length ($L_{FBG}$) is directly proportional to grating peak reflectivity but inversely proportional to linewidth of the grating, according to Equation (4),

(4) $$Linewidth=\lambda_{Bragg}S\sqrt[{}]{{\frac{\Delta n}{2n_{eff}}}^{2}+{\frac{\lambda }{L_{FBG}}}^{2}},$$

where $\lambda_{Bragg}$ is the Bragg wavelength, *S* is a constant describing reflective strength, Δn is the RI modulation of the grating fringe, $n_{eff}$ is the effective index of the fiberand Λ is the pitch of the grating. Hence, Sensor B with a grating length of 6 mm has a significantly narrower back reflection linewidth at each peak as compared to Sensor A with a short 1 mm grating length, resulting in a difference in the observed spectra output. The interference at each of the reflection peaks observed in Figure 2 can be expressed as:

(5) $$I^{m}=I_{R1}+I_{R2^{m}}+2\sqrt[{}]{I_{R1}+I_{R2^{m}}}cos\delta ,$$

where $I^{m}$ is the interference intensity at peak m, $I_{R1}$ is the intensity of the broadband light reflected by the fiber end and $I_{R2^{m}}$ is the intensity of the reflected light at each SSFBG peak. δ is the phase difference between $I_{R1}$ and $I_{R2^{m}}$ and is given by:

(6) $$\delta =\frac{4\pi n_{eff}L}{\lambda },$$

where $n_{eff}$ is the effective index of the optical fiber at the operating wavelength, *L* is the cavity length and λ is the operating wavelength. It is then apparent that the length *L* of the cavity is inverse in relation to the free spectra range (FSR) of the resulting interference peaks. To obtain a larger FSR for good resolvability of the spectrum, a short cavity length is preferred.

### 2.4. Sensing Scheme

As the fiber end is exposed to substances of different RI, Fresnel reflection from the cleaved end varies according to Equation (7) while reflection from the SSFBG remains unchanged:

(7) $$I_{R1}=I_{ASE}\frac{{\left(n_{eff}-n_{ambient}\right)}^{2}}{{\left(n_{eff}+n_{ambient}\right)}^{2}},$$

where $I_{ASE}$ is the intensity of the ASE light source, $n_{eff}$ is the effective index of the optical fiber at the operating wavelength and $n_{ambient}$ is the ambient RI. This results in a change in visibility (*v*) of FP interference at each peak. To have a fixed reference, visibility here is calculated from the center fringe peak subtracting the right immediate valley in the FPI. This change is then a direct implication of the change in ambient RI as explained previously. The need for lower reflectivity peaks (i.e., $R2^{-1}$) arises as R1 decreases to such a low intensity that the visibility of the higher reflecting peaks (i.e., $R2^{0}$) would not be resolvable due to the large intensity difference between $I_{R1}$ and $I_{R2^{m}}$. In contrast, an R1 matching reflection (i.e., $R2^{0}$) from a single FBG will not be suitable for measurement due to the small unresolvable visibility when the ambient RI approaches that of the fiber effective index.

### 2.5. Chelating Agent for the Formation of Metal Chelates

**Figure 3:**
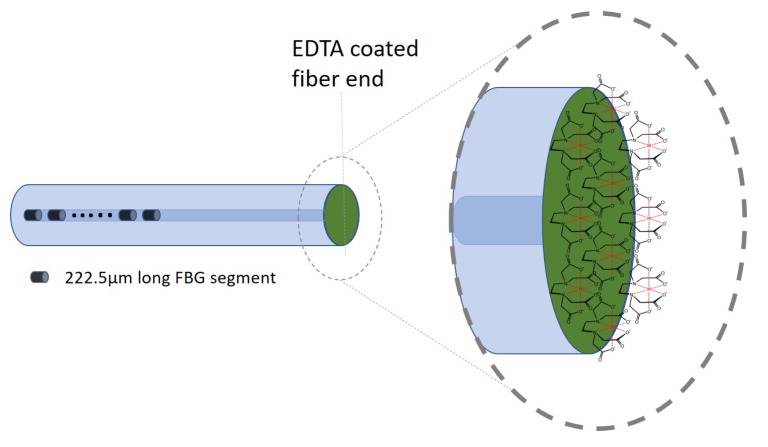
Ethylenediaminetetraacetic acid coated fiber end.

Chelating agents (or chelators) are defined as multidentate ligands whose structures are capable of binding donor atoms (or sites) to metal ions, producing an extremely stable, complex ring-like structure called metal chelates [26]. These chelators are usually comprised of a chelating ligand that consists of binding atoms in the form of chemical groups like –SH, –*S*–*S*, –$NH_{2}$ , =NH, –OH, –$OPO_{3}H$ or >*C*=*O* to achieve metal binding capabilities [27]. In this work, a universal metal chelator, namely EDTA, was chosen as a coating to be coated onto the fiber end as shown in Figure 3.

The hexadentate ligand (i.e., six potential binding sites) in EDTA, which consists of four carboxylate and two amine groups [28], would serve as binding points for metal ions such as cadmium (Cd${}^{2+}$) and lead (Pb${}^{2+})$. Chelation properties of EDTA with different metal ions depend on its stability constant(k) [27]. Metal with higher k constant competes and displace metal ions of a lower k constant. Heavier metal ions tend to have a much higher constant and hence forms stable chelate. Thus, functionalizing a chelating agent on the tip of the fiber will allow these heavy metal ions to be captured at the fiber tip. This results in a change in RI of the EDTA that would modulate the light interacting at the fiber tip.

## 3. Sensor Fabrication and Experiment

### 3.1. Superstructure Bragg Grating Inscription

**Figure 4:**
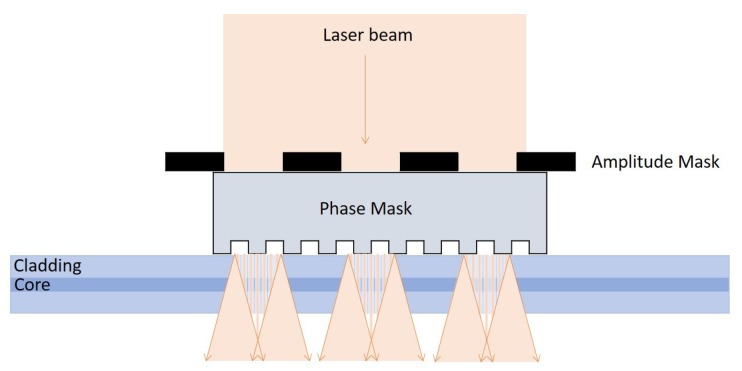
SSFBG fabrication method.

The fabrication of the sensors is a two-step process. For sensor A, a 1 mm SSFBG was inscribed onto a short segment of the photosensitive PS1250 single mode fiber. For sensor B, a 6 mm SSFBG was inscribed onto the UHNA 7 single mode fiber. The SSFBG inscription was carried out using a 244 nm laser with a spot size of 1 mm. Both fibers were exposed to the laser through a mask system consisting of a 545 μm pitch amplitude mask placed 4 mm in front of a phase mask grating plane of 1075.2 nm pitch, as illustrated in Figure 4.

The laser beam was translated 1 mm for sensor A and 6 mm for Sensor B across the mask system to inscribe a SSFBG with a length of 1 mm and 6 mm for Sensors A and B, respectively. The transmission spectrum was monitored and the exposure was terminated once the reflectivity of the center peak reached approximately 4% for both Sensors A and B. The fibers were then cleaved at a distance of 5 mm for Sensor A and 30 mm for Sensor B away from the center of the SSFBG.

**Figure 5:**
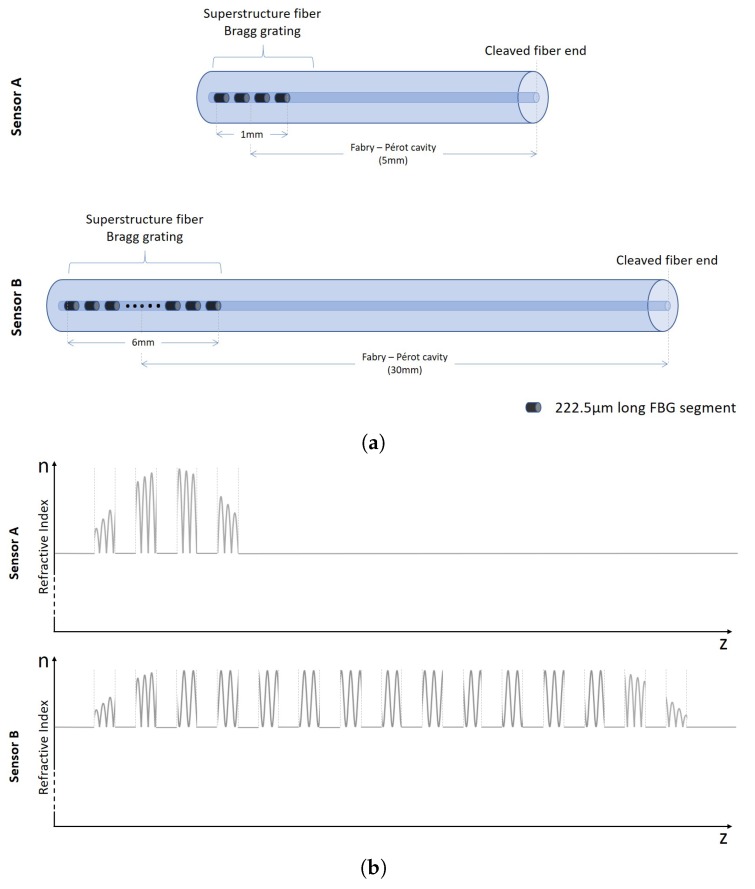
Sensor schematics diagram of (**a**) sensor configuration; and (**b**) refractive index modulation resulting in sensor SSFBGs.

Cavity length of the two sensors was determined after considering various factors. A shorter length is preferred for the compactness of sensor so that the sensing probe need not be submerged deeper into the solution. As the linewidth of the SSFBG in sensor A is larger, a shorter cavity length would be advantageous due to the resulting larger FSR of the interference pattern. In contrast, the narrow linewidth of SSFBG in sensor B limited the magnitude of the FSR, hence the much longer cavity design. However, there is also a practical limit to short cavity design as cleaving at a distance too short is challenging and there is a risk of damaging the grating. The resulting sensors are depicted in Figure 5. Sensor A is made up of 4 short 222.5 μm long FBGs while Sensor B is made up of 15 short 222.5 μm long FBGs.

Figure 5b is a representation of the index modulation along the length of the sensor fiber resulting in the SSFBG structure. The periodic index modulation is through UV irradiation using a phase mask, further masked with a amplitude mask. Behavior of both sensors to variations in RI were then characterized (see Figure 8) and results discussed in Section 4.1.

### 3.2. EDTA Coating for Heavy Metal Sensitivity

The cleaved ends of both fibers were first dipped into acetone to remove dirt and dust particles. They were then immersed in Piranha solution (a mixture of sulfuric acid and hydrogen peroxide at 3:1 *v*/*v* ratio) for 30 min inside a fume hood. The fiber ends were then thoroughly rinsed with deionized (DI) water and dried using a nitrogen gun. They were next silanized using a 2% *v*/*v* of 3-aminotrimethoxysilane in toluene for 90 min at 70 ${}^{\circ }$C. Any weakly binding amine groups were then removed through rinsing with ethanol and drying in a furnace at 80 ${}^{\circ }$C for 90 min. The coating was prepared by mixing 20 mg of EDTA with 15 mL of phosphate buffer saline solution. In addition, 6 mg of 1-ethyl-3-(3-dimethylaminopropyl) carbodiimide hydrochloride (EDC, (crosslinker)) was added to activate the carboxyl groups of the EDTA solution. Furthermore, 10 mg of *N*-hydroxysuccinimide (NHS) was also added as a stabilizer and the resulting solution was left undisturbed for 15 min at room temperature. The silanized fiber tip was dipped into the activated chelating agent and left undisturbed for 2 h to allow covalent bonds to form. 10 mg of hydroxylamine was finally added to the solution to stop the reaction.

## 4. Experiment and Results

**Figure 6:**
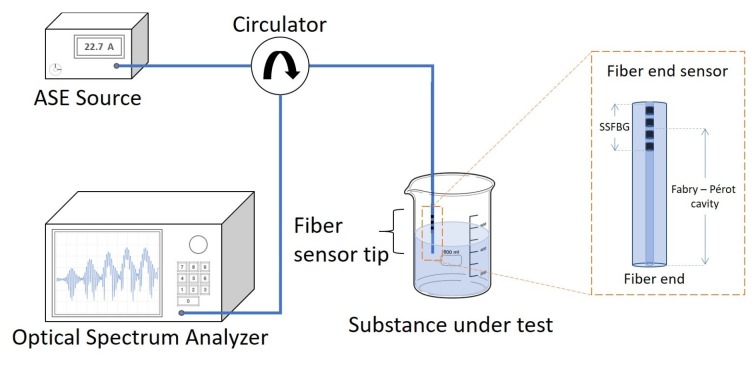
Experimental setup.

Each sensor was connected to a broadband light source and an optical spectrum analyzer (Model: ANDO AQ6317B; Wavelength resolution: 0.01 nm; Power resolution: 0.01 dB) using a circulator as shown in Figure 6. For RI sensing, the fiber tip was immersed in liquids with RIs ranging from 1.333 to 1.470. The depth of fiber immersion depends on the information of the liquid that is desired. For example, if only information of the RI of the liquid is to be measured, then only the cleaved end needs to be submerged. Otherwise, if the temperature of the liquid is to be measured, then the SSFBG must be submerged in the liquid as well.

### 4.1. Refractive Index Characterization

To characterize the sensor for RI measurement of air and from 1.333 to 1.470, 14 liquids with different RIs were prepared using Glycerine in increments of 0.010 refractive index unit (RIU). The sample RI were measured with a refractometer (KRUSS GmbH DR201-95, (Hamburg, Germany)) with $10^{-4}$ accuracy. After each measurement, the sensor was left to sit in a large beaker of 1 L of deionised water for 1 min and flushed with deionised water before the next measurement.

**Figure 7:**
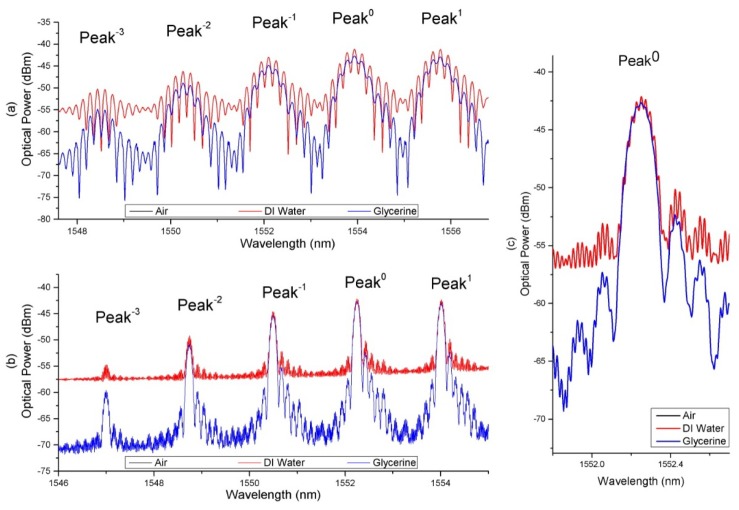
(**a**) interference spectrum of sensor A with cleaved end interfaced deionised water and Glycerine solution of 1.433 RIU; (**b**) interference spectrum of Sensor B with cleaved end interfaced with deionised water and Glycerine solution of 1.470 RIU; (**c**) expanded view on peak 0 of Sensor B.

Figure 7 shows the resulting spectrum at the reflection port of the circulator when the sensors were exposed to DI water and Glycerine solution with a RI 1.433 for Sensor A (Figure 7a) and 1.470 for Sensor B (Figure 7b). For ease of explanation, we have numbered the peaks with respect to the center peaks. Center peaks are at 1554.1 nm and 1552.3 nm for Sensors A and B, respectively.

**Figure 8:**
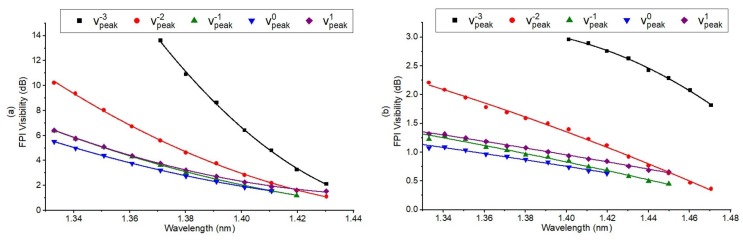
(**a**) Fabry–Perot interference visibility for Sensor A at each peak of SSFBG when exposed to ambient refractive index in the range of 1.333 to 1.433; (**b**) Fabry–Perot interference visibility for Sensor B at each peak of SSFBG when exposed to air and ambient refractive index in the range of 1.333 to 1.470.

The relationship between the visibility of the peaks with the RI of the various Glycerine solutions is plotted in Figure 8, which shows a clear relationship between the visibility of the peaks and the RI of the solutions. Visibility is taken with respect to the largest fringe contrast in the middle of envelope of each reflection reflection peak. As the ambient RI increases, visibility of the FP interference formed at each SSFBG peaks decreases and vice versa.

Each SSFBG peak of a sensor is sensitive only within a particular RI range. For example, in Sensor B, the interference spectrum at peak 0 ceased to be resolvable from an RI of 1.433 upwards due to large reflectivity mismatch between the SSFBG reflection of peak 0 and the fiber end reflection. At this level of ambient RI, the fiber end reflectivity for sensor B is close to 0.4%, significantly lower than the SSFBG peak 0 reflectivity of 3.7%. Therefore, the SSFBG multi reflection peak at decreasing reflectivity enables the same fiber to remain sensitive in a wider RI range. From Figure 7, the working range for the sensors, determined by the range of RI where the visibility of the FP interference is resolvable at one or more of the SSFBG peaks is 1.333 to 1.433 with maximum sensitivity of approximately 230 dB/RIU, resulting resolution of $4.35\times 10^{-5}$ RIU (RI range of 1.370 to 1.390) and 1.333 to 1.470 with maximum sensitivity of 24 dB/RIU, resulting resolution of $4.17\times 10^{-4}$ RIU (RI range of 1.433 to 1.460) for Sensors A and B, respectively. Sensitivity of a fiber end sensor is limited by light ambient interaction area at the fiber end. In our sensors, this area is determined by the MFD of the fundamental guided mode of the host fiber. Sensor B hosted on UHNA 7 with high NA and small core naturally resulted in a much smaller MFD as compared to Sensor A that was designed for MFD matching with standard single mode fiber (i.e., SMF28e). Despite the larger sensing range for Sensor B, Sensor A is a better candidate for its higher sensitivity within its working RI range.

### 4.2. Heavy Metal Detection Response

**Figure 9:**
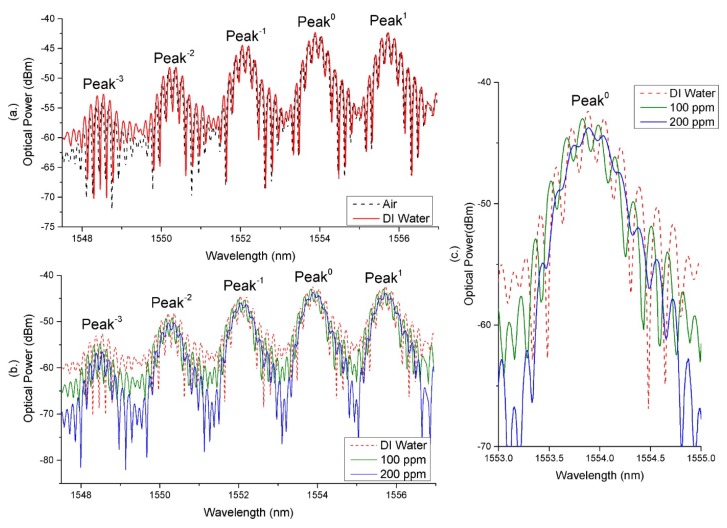
(**a**) Ethylenediaminetetraacetic acid coated sensor A response when exposed to air and in deionized water; (**b**) Ethylenediaminetetraacetic acid coated Sensor A response when exposed to deionized water water and in 100 ppm and 200 ppm solution of Cd${}^{2+}$; (**c**) enlarged view of Peak 0.

The EDTA coating was applied to both sensors using the process described in Section 3.2 and the fiber sensors were found to be insensitive to variations in ambient RI as shown in Figure 9a. As can be seen from the figure, the reflected spectrum remains the same when the sensor was placed in air and in DI water. This shows that the thin layer of EDTA insulated the fiber end from the ambient environment. The RI of the coating of EDTA without heavy metal chelate is found to be 1.356. As part of the surface functionalization process, the coated fiber end was left to sit in the DI water over night so as to ensure that EDTA was indeed properly adhered to the fiber end surface.

When placed into DI water doped with 200 ppm of Cd${}^{2+}$ ions for 10 s, a change in the fringe visibility could be observed for both sensors. This evidently demonstrates the ability of the coated sensor to detect Cd ions at the ppm level. However, only Sensor A’s response was conclusive. As Sensor B’s MFD is too narrow, it was found that sensitivity to heavy metal ion is greatly compromised as too few heavy metal ion capturing sites falls within the light interaction area at the fiber end.

A further experiment for heavy metal ion detection was conducted with only Sensor A using a waste water sample received from a local water agency in Singapore. These samples were DI water doped with 1 ppm, 10 ppm, 100 ppm and 200 ppm of Cd${}^{2+}$ ions, and it was found that 10 ppm was the minimum detectable concentration that would cause a significant change in RI of the EDTA coating when the sensor was allowed to sit in the solution for 10 s with observable visibility change. However, this measurement is not sufficiently conclusive as it falls near the error range of the measurement equipment. Hence, a significantly conclusive measurement can only be achieved from the sample with concentration of 100 ppm. This is shown in Figure 9b where the visibility of the FP interference fringes decrease significantly after the sensor tip was immersed in solution of 100 ppm and decreases further in 200 ppm Cd${}^{2+}$ ions for just 10 s in each sample.

Sensor A’s sensitivity to Cd${}^{2+}$ ions in the range of under 100 ppm was then found to be 0.150 dB/10 ppm (resolution: $6.67\times 10^{-2}$ per 10 ppm) of Cd${}^{2+}$ ions. From 100 ppm to 200 ppm, it was found that the sensitivity is 0.143 dB/10 ppm (resolution: $6.99\times 10^{-2}$ per 10 ppm) of Cd${}^{2+}$ ions. This is to say, industrial waste would have reached the fatal level of Cd${}^{2+}$ concentration if visibility of Sensor A decreases by more than 1.5 dB taking reference from visibility of sensor when in DI water.

### 4.3. Temperature Response of EDTA Coated Fiber Sensor

**Figure 10:**
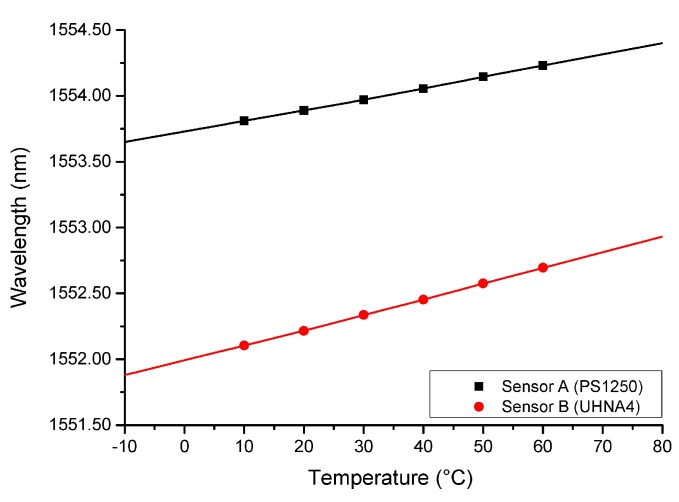
Plot of Sensor A and B’s central peak Bragg wavelength shift against temperature variation.

The ambient temperature is also an important parameter in RI measurements as the ambient RI is cross-related to its temperature. The behavior of both sensors to variations in temperature were observed by immersing the sensors into a water bath at different temperatures. Figure 10 charts the temperature to Bragg wavelength relationship of both Sensor A and Sensor B. Plotted values were taken as the average of three data sets for each fiber. It is noteworthy that, with sufficient time allowance (>30 s) between each test for the fiber to achieve temperature of the water bath, the three data sets are largely identical, limited only by the resolution of the optical spectrum analyzer.

The experiment was conducted in the reasonable range of operating temperature in an increment of 10 degrees from 10 to 60 degrees, 10 degrees lower and 10 degrees higher than reasonable temperature of a water body in industrial plants. As expected from a silica FBG temperature sensor, temperature response is largely linear in a small temperature range. In addition, the property of silica fiber results in no observable hysteresis as deformation within the tested temperature range will not cause significant permanent deformation to sillica fibers. The temperature sensitivity is found to be 8.5 pm/${}^{\circ }$C and 12.0 pm/${}^{\circ }$C for Sensor A and Sensor B, respectively. The difference in temperature sensitivity can be attributed to the difference in fiber core dopant material and concentration.

As temperature measurement is by the shift of Bragg wavelength according to Equation (3) discussed in Section 2.2, independent from RI measurement using visibility of FP interference formed by the grating and cleaved fiber end, measurement of the temperature and RI can then be carried out simultaneously.

## 5. Conclusions

We have demonstrated two superstructure Fiber Bragg grating (SSFBG) fiber end refractive index (RI) sensors based on Fabry–Perot interference formed between the SSFBG and the cleaved end of the fiber. The effective index of each fiber limits the upper RI sensing range of the sensors. The sensors can be designed to be sensitive to variations in ambient RI with RIs ranging from 1.333 to 1.470. RI information can be extracted from the visibility of the reflected light of both sensors. Building on the fiber RI sensors fabricated, we have demonstrated an application as a heavy metal ion detector when a coating of Ethylenediaminetetraacetic acid was applied. The sensors were also capable of simultaneous measurement of temperature using Bragg wavelength shift information, due to the fact that RI is measured from fringe visibility of FP interference spectra, independent of the Bragg wavelength shift. This SSFBG-FPI sensor is a good alternative to other sensing schemes that have already been proposed. Interrogation of this sensor is simpler as compared to tilted FBG sensors with comparable sensitivity and working range limited only by the doping of the host fiber. The physical robustness of this sensor is higher then cladding removed or tapered FBG sensors. A higher sensitivity can be achieved as compared to noise error prone FBG to fiber end reflection ratio-meters. EDTA deposition on silica fiber was also demonstrated as a alternative coating for fiber end sensors for heavy metal chelation. 

## Abbreviations

The following abbreviations are used in this manuscript:

FBG	Fiber Bragg grating
RI	Refractive index
RIU	Refractive index unit
SSFBG	Superstructure fiber Bragg grating
